# Transforming Hospital Care: Impact of an Evidence‐Based Practice Course on Healthcare Professionals' Competencies in a Randomized Clinical Trial

**DOI:** 10.1111/wvn.70130

**Published:** 2026-04-01

**Authors:** Elaine Cristina Marqueze, Heidy Oliveira Portela, Evelyn Carla Borsari Mauricio, Cristina Satoko Mizoi, Maiana Regina Gomes de Sousa, Sara Becker Oliveira, Marinete Esteves Franco

**Affiliations:** ^1^ Hospital Nove De Julho São Paulo SP Brazil; ^2^ Federal University of Rio Grande Do Norte (UFRN) São Paulo RN Brazil

**Keywords:** controlled clinical trials, educational intervention, evidence‐based practice, healthcare professionals, hospital, randomized

## Abstract

**Introduction:**

Evidence‐Based Practice (EBP) is essential to healthcare quality and safety, integrating scientific evidence with clinical expertise and patient preferences. Despite its importance, EBP implementation still faces major challenges. Educational interventions have proven effective in strengthening EBP competencies among healthcare.

**Main:**

To evaluate the impact of a personalized educational intervention on EBP competencies among healthcare professionals. Working at a private tertiary general hospital, comparing performance before and after the intervention.

**Methods:**

A randomized controlled trial involving healthcare professionals was conducted. Eligible and consented participants were randomly assigned to either an Intervention Group (IG) receiving an Evidence‐Based Practice (EBP) course or a Control Group (CG) not receiving the course, stratified by job level, role, and work shift. From the completers, 18 participants were randomly selected for the IG, and all 7 available CG participants were included in the final sample for analysis. All study participants completed two validated instruments: the Assessing Competencies in Evidence‐Based Medicine (ACE) and the Fresno Test. The educational intervention consisted of a seven‐week course with weekly three‐hour sessions, for a total of 21 h. Comparative analyses were conducted using a Linear Mixed Model, adjusted for educational level, job level, time working at the hospital, and weekly workload.

**Results:**

A statistically significant increase in general EBP knowledge was observed in the IG following the intervention, with a mean gain of 19.1%. Separate analysis showed improvements of 10.8% in ACE and 24.2% in Fresno Test scores. No statistically significant changes were observed in the CG. Furthermore, after the intervention, the IG outperformed the CG for both general EBP knowledge and Fresno Test scores on both pre‐ and post‐intervention comparisons.

**Conclusion:**

The educational intervention had a positive statistically significant impact on EBP knowledge and skills among healthcare professionals in the IG compared to the CG. These findings underscore the potential of structured educational initiatives to enhance the quality of clinical practice through improved EBP competencies.

**Trial Registration:**

UTN U1111‐1322‐8443

## 1 Introduction

Evidence‐Based Practice (EBP) represents both a conceptual and practical evolution of Evidence‐Based Medicine (EBM), broadening its scope to encompass interprofessional teams, patient values, preferences, and circumstances, as well as the best available evidence in clinical decision‐making. Originally conceived as a clinical learning approach at McMaster Medical School, EBP has become an essential paradigm for enhancing healthcare quality and safety, ultimately optimizing patient outcomes. The traditional EBP process entails five steps–Ask, Acquire, Appraise, Apply, and Assess–that aim to improve patient outcomes. Recent developments, however, have led to the incorporation of additional stages, such as fostering an investigative mindset and disseminating findings, thereby expanding its application and encouraging a more critical and reflective practice (Dawes et al. 2005).

Despite evidence demonstrating that EBP improves clinical outcomes, its integration into professional practice continues to face significant challenges. Barriers such as limited time, insufficient organizational support, and gaps in research‐related skills have hindered the routine adoption of EBP in clinical settings. Furthermore, many healthcare professionals were not exposed to the EBP paradigm during their initial training, complicating efforts to fully embrace this approach (Jeong et al. 2024).

In this context, educational interventions have emerged as a promising strategy to empower professionals and overcome these obstacles. Studies suggest that well‐structured EBP education can enhance knowledge and skills, while also influencing attitudes and fostering a culture of inquiry and continuous learning. Nonetheless, there is still no consensus on the most effective methods for teaching and integrating EBP into practice (Melnyk et al. 2014).

Although teaching strategies for EBP vary, multifaceted interventions‐combining lectures, practical sessions, journal clubs, and problem‐based learning–have proven more effective than single‐method approaches. The integration of EBP as an interdisciplinary competency is widely recommended, emphasizing the importance of developing critical thinking and information literacy from the outset of professional training. Additionally, proficiency in EBP is recognized as essential in health professional education, necessitating the development of validated tools for assessing knowledge, skills, and attitudes in this area. Nevertheless, gaps remain in the literature regarding the optimal duration of interventions, most effective methodologies, and best instruments for evaluating educational outcomes in EBP (Melnyk et al. 2014; Ruzafa‐Martínez et al. 2016).

### 1.1 Purpose and Aims

The aim of the present study was to evaluate the impact of a personalized professional educational intervention on Evidence‐Based Practice (EBP) competencies among healthcare professionals in a private tertiary general hospital, assessed before and after the intervention. The specific objectives were: 1. To assess whether the intervention led to improvements in EBP competencies, in terms of overall knowledge of EBP, as well as performance on the ACE and Fresno tests individually, in both the intervention and control groups; 2. To analyze interactions between groups (intervention versus control) and time points (pre‐ and post‐intervention) in terms of overall EBP knowledge, as well as ACE and Fresno Test results; 3. To explore participant perceptions of the course and its applicability in developing EBP projects.

## 2 Design and Methods

The research project was submitted to and approved by the Research Ethics Committee and was carried out in accordance with the Declaration of Helsinki. The clinical trial protocol is registered on the World Health Organization's International Clinical Trials Registry Platform. The study was conducted in accordance with the CONSORT guidelines for non‐pharmacological trials–CONSORT‐NPT (Boutron et al. 2017).

A Randomized Controlled Trial (RCT), non‐intervention control group clinical trial involving healthcare professionals working at a private tertiary general hospital was conducted. The study aimed to evaluate an Evidence‐Based Practice (EBP) course. A pool of eligible professionals from the hospital staff was identified during the first half of 2025. All participants met the study eligibility criteria. Prior to the course, participants received an email invitation outlining the study objectives. The email explained that participants could be randomized as part of the study (either an Intervention Group‐IG or a Control Group‐CG) and that, on the first day of the course, they would be asked to complete the EBP competency assessment tests and sign the Informed Consent Form (ICF).

All individuals invited agreed to participate in the study. On the first day of the course, participants provided their names and email addresses to be included in the randomization process and signed the ICF, with assurance of confidentiality regarding their personal data. For the Control Group, 22 healthcare professionals on the hospital staff who met the same eligibility criteria as those in the Intervention Group (*n* = 36) in terms of job level, role, and work shift were selected. These professionals also received an email invitation explaining that they would be part of the CG. This group attended the first day of the course and, alongside the IG, completed the EBP competency assessment tests and signed the ICF. All participants were allotted 60 min to complete the assessment. Upon completion of the tests, CG members were dismissed, and the course commenced for the IG.

The sample size was calculated a priori based on results reported by Tucker et al. (2022), who examined the effects of an intervention on EBP attributes (knowledge, beliefs, and competence). Mean values for EBP skills at baseline (T0) and post‐intervention were used for sample size estimation, aligning with the objectives of the present study.

The effect size ‘*d*’ reported by Tucker et al. (2022) (*d* = 1.538) was converted into the effect size ‘*f*’ using an online tool (https://www.psychometrica.de/effect_size.html). The parameters applied in the G*Power software for the repeated measures ANOVA (within‐between interaction) were: effect size *f* = 0.769, two groups (intervention and control), two time points (pre‐ and post‐intervention), alpha = 0.05, and sample power = 0.99 (beta error). The estimated sample size was 18 participants. Considering a potential 40% attrition rate, the target sample size was adjusted to 25 participants.

After the intervention, all members of the IG who completed the EBP course (*n* = 36) and CG (*n* = 22) were asked to complete the EBP competency assessment tests again. The post‐intervention assessments were administered on the final day of the course, after all content had been delivered. All IG participants (*n* = 36) completed the assessments, while only seven individuals from the CG returned for the post‐test. The time allocated for the assessment was unchanged at 60 min.

**FIGURE 1:**
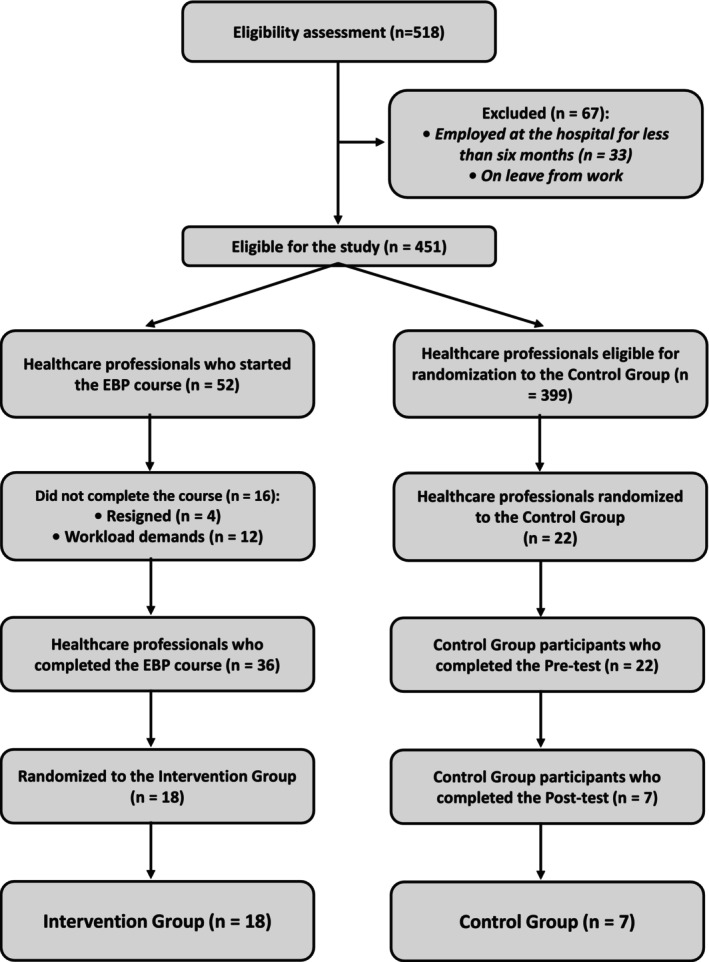
Study flowchart.

To reduce potential confounding, participants in both IG and CG were stratified by job level, role, and work shift before random selection. From the 36 IG course completers, 18 participants were randomly selected to match the final sample size required for analysis. For the CG, all seven CG participants who completed both the pre‐ and post‐intervention assessments were included in the final sample, resulting in a total of 25 individuals in the final sample. The resultant distribution precluded group pairing for the final analysis. The study flowchart is presented in Figure 1.

To evaluate whether attrition in the CG introduced bias or compromised the validity of the findings, sociodemographic (age, marital status, education) and professional variables (tenure at the institution and in the sector, profession, work shift, job level, and work area) of the final CG participants (*n* = 7) were compared against those of the 11 individuals who had initially agreed to participate in the CG. No statistically significant differences (*p* > 0.05) were found between these groups, suggesting that the attrition was random and did not introduce systematic bias into the study.

Two validated instruments were used to assess EBP competencies: (1) the Assessing Competency in Evidence‐Based Medicine (ACE) tool; and (2) the Fresno Test. Different clinical scenarios were used in both pre‐ and post‐intervention assessments to minimize recall bias and ensure that improvements reflected genuine skill development rather than memorization.

The ACE tool, developed and validated by Ilic et al. (2014), presents participants with a clinical scenario, a structured clinical question, a search strategy, and a summary of a research article. Subsequently, 15 dichotomous (‘yes’ or ‘no’) questions are posed covering four of the five steps of EBP: formulating clinical questions (items 1–2), searching scientific literature (items 3–4), appraising the evidence (items 5–11), and applying evidence to clinical practice (items 12–15). In this study, the Portuguese version of the ACE validated by Maia et al. (2022) was employed. Scores were calculated based on the percentage of correct answers, with each correct response contributing equally to a total possible score of 100%. In other words, each question represented a fraction of the total, and the final score was expressed as a percentage, making it easier to compare.

The Fresno Test, originally developed at the University of California, San Francisco, in 2003, is a performance‐based assessment tool used to evaluate EBP knowledge among students, educators, and healthcare professionals (Salerno et al. 2019; Ramos et al. 2003). The test presents two clinical scenarios involving diagnostic or therapeutic uncertainty, followed by nine open‐ended questions. Topics covered include study design, database selection, types of studies, search strategies, relevance and validity of findings, magnitude and significance of results, sensitivity and specificity, predictive values, probability, number needed to treat, confidence intervals, diagnosis, and prognosis. The Portuguese version validated by Salerno et al. (2019) was used in this study. Akin to the ACE, scores on the Fresno Test were expressed as percentages.

Given that the ACE tool emphasizes practical EBP competencies, while the Fresno Test assesses broader theoretical knowledge, both instruments were used concomitantly to provide a more comprehensive evaluation of participant skills and understanding. An overall EBP knowledge score was calculated by averaging the percentage scores for the two instruments, ensuring balanced representation of performance on both tests. Test corrections were performed blindly by the course instructor to avoid potential bias.

Also, participants were asked to evaluate the quality of the course materials, teaching methods, course duration, clarity of content, and relevance of the training to EBP project development. This evaluation used a five‐point Likert scale and was designed by the researchers. In addition to the competency assessments, participants completed a sociodemographic and occupational questionnaire designed by the researchers to characterize the sample.

The EBP course spanned seven weeks, consisting of weekly sessions lasting three hours each, for a total duration of 21 h. The course structure was guided by the meta‐analysis conducted by Jeong et al. (2024), which evaluated the effectiveness of educational programs in evidence‐based practice. According to their findings, programs with a total duration of 20–24 h, delivered over four to seven weeks, yielded the most significant improvements in EBP skills, critical thinking, and problem‐solving abilities.

The course was given by the study's lead author, a PhD holder in Public Health, and was designed to be interactive, incorporating practical exercises and knowledge assessment tasks. Participants who achieved a minimum score of 70% on the assessments and attended at least 70% of the sessions received a certificate of completion.

The course syllabus, also developed by the author based on extensive academic and professional experience, included the following topics:

1. Evidence‐Based Practice: definition, historical background, importance of EBP, and related practical exercises.

2. Search Strategies (MeSH/DeCS, Boolean Operators, Delimiters): techniques for constructing research questions (PICO, PECO, PEO, PICo), keyword selection, and literature searches using open‐access materials from Johns Hopkins.

3. Scientific Databases: PubMed, Scopus, Cochrane, UpToDate, Mendeley, Periódicos CAPES, ResearchGate; evaluation of journal quality, identification of predatory journals, reference management tools, and strategies for accessing restricted articles.

4. Review and Epidemiological Studies: types of literature reviews (scoping, narrative, integrative, systematic, meta‐analyses) and study designs (observational and clinical studies), limitations, evidence appraisal tools, and guided practical sessions.

5. Quality of Evidence: concepts of validity and reliability, internal and external validity, internal consistency, statistical significance, confidence intervals, sensitivity and specificity, predictive values, likelihood ratios, risk measurements, and use of checklists and reporting guidelines for scientific writing.

6. Application of EBP in Practice (PET Process – Practice question, Evidence, Translation): decision‐making based on evidence, feasibility and acceptability assessments, implementation strategies, and evaluation through performance indicators.

The aim of the educational intervention was to contribute to the development of more effective training strategies tailored to the needs of healthcare professionals. Furthermore, the study sought to promote the integration of EBP into clinical practice, encouraging the provision of care that is not only evidence‐informed but also aligned with the principles of patient‐centered care. This approach is crucial for strengthening healthcare quality and addressing the evolving challenges of modern clinical environments.

### 2.1 Inclusion Criteria

Participants were healthcare professionals holding higher education degrees who had been working at the hospital for at least six months. The IG included only individuals that had never participated in an Evidence‐Based Practice (EBP) course.

### 2.2 Exclusion Criteria

Exclusion criteria for the IG comprised participants that failed to achieve a minimum 70% attendance in the course or who were concurrently enrolled in another EBP course during the study period.

### 2.3 Data Analysis

A descriptive analysis of the study variables was performed, with categorical variables expressed as absolute and relative frequencies, and continuous variables as measures of central tendency and dispersion. Sociodemographic and work‐related data for the control group (CG) and respective losses were compared using Student's *t*‐test for independent samples or the Mann–Whitney U test for quantitative variables, according to data distribution. while the chi^2^ or Fisher's exact proportion tests were used for qualitative variables.

Pre‐ and post‐intervention time points were compared using a Linear Mixed Model (LMM), which incorporates a random intercept for each participant. This approach allowed inter‐individual variability to be captured, such as professionals who began the course with already high or low levels of knowledge about EBP, regardless of the intervention. Inclusion of a random effect for each subject addresses the assumption of independence among observations, considering the natural correlation between repeated measurements within the same individual, and enhances the precision of fixed effect estimates, such as the effect of the intervention.

Separate models were developed for the IG and for the CG, while an interaction model involving both the IG and CG was also constructed. The model was adjusted for variables including educational level, job level, time working at the hospital, and weekly workload. This comprehensive approach aimed not only to assess the effectiveness of the intervention but also to control for potential confounding factors that could influence the acquisition of competencies in EBP. A significance level of *p* < 0.05 was adopted, and all statistical analyses were conducted using Jamovi software, version 2.6.13.0.

## 3 Results

### 3.1 Description of Control Group (CG) and Intervention Group (IG)

Both the Control Group (CG) and IG comprised predominantly female participants (85.7% and 88.9%, respectively). There were no statistically significant differences between the groups in terms of mean age, marital status, or educational level. Participants in both groups were mature adults, with a predominance of nurses holding a graduate degree (Table 1).

**TABLE 1:** Sociodemographic and work variables of healthcare professionals (*n* = 25).

Variables	CG	IG	*p*
	*n* (%)	*n* (%)	
**Mean age (years)**	43.9 (6.0)	40.0 (6.5)	0.18^a^
**Marital status**			
Married/Civil union	3 (42.9)	10 (55.6)	
Single	3 (42.9)	5 (27.8)	
Separated/Divorced	1 (14.3)	2 (11.1)	
Widowed	0 (0)	1 (5.6)	0.88
**Educational level**			
Undergraduate degree	0 (0)	1 (5.6)	
Graduate degree	7 (100)	13 (72.2)	
Master's degree–complete	0 (0)	1 (5.6)	
Master's degree–incomplete	0 (0)	1 (5.6)	
PhD. complete	0 (0)	2 (11.1)	1.00
**Professional role**			
Nurse	6 (85.7)	15 (83.3)	
Physician	0 (0)	2 (11.1)	
Pharmacist	1 (14.3)	1 (5.6)	1.00
**Work shift**			
Day shift	7 (100)	12 (66.7)	
Morning shift	0 (0)	4 (22.2)	
Evening shift	0 (0)	1 (5.6)	
Night shift	0 (0)	1 (5.6)	0.63
**Job level**			
Mid‐level	3 (42.9)	5 (27.8)	
Senior	1 (14.3)	5 (27.8)	
Coordinator	3 (42.9)	4 (22.2)	
Manager	0 (0)	4 (22.2)	0.47
**Mean time working at the hospital (years)**	9.3 (11.6)	5.8 (5.4)	0.79^b^
**Mean time working in current units (years)**	9.3 (11.6)	5.2 (5.4)	0.88^b^
**Mean weekly workload (hours)**	43.6 (1.6)	41.6 (5.5)	0.16^b^

^a^ Student's *t*‐test for independent samples.

^b^ Mann‐Whitney *U* test for independent samples.

The majority of participants worked regular shifts and held senior or full‐time positions, with no significant group differences for these aspects. Additionally, there were no statistically significant differences between the CG and IG regarding mean time working at the hospital, time working in current units, or mean weekly workload (Table 1). The work units were diverse and outlined in more detail in Table 1.

### 3.2 Assessment of EBP Competencies

On the analysis of the Control Group (CG) using the Linear Mixed Model (LMM), adjusted for educational level, time working at the hospital, and weekly workload, no statistically significant effect was observed on the general knowledge in Evidence‐Based Practice (EBP) score following the intervention, compared to baseline. This composite score included results from both the ACE and Fresno tests. Furthermore, the absence of a statistically significant effect in the CG persisted when analyzing each instrument separately, i.e., EBP competencies in the CG remained unchanged throughout the follow‐up period (Table 2).

**TABLE 2:** Linear mixed model for general knowledge in evidence‐based practice (EBP) score, ACE test, and Fresno test, pre‐ and post‐intervention in control group (*n* = 7).

Variables	Mean (SD)	95% CI	% difference	F (df)	*p*	Model Fit	*R* ^2^ (df)	LRT *X* ^2^	*p*
General score (0%–100%)*									
Pre	28.1 (12.8)	16.2–40.4	−0.1	0.0008 (1)	0.98	Conditional	0.510 (6)	13.9	0.031
Post	28.0 (11.8)	17.1–38.8				Marginal	0.510 (5)	12.8	0.025
ACE test (0%–100%)*									
Pre	56.2 (19.2)	38.4–73.9	0.0	0.000000002 (1)	1.00	Conditional	0.207 (6)	5.0	0.55
Post	56.2 (14.8)	42.5–69.9				Marginal	0.207 (5)	5.0	0.42
Fresno Test (0%–100%)*									
Pre	11.2 (11.6)	0.5–22.0	−0.2	0.007 (1)	0.94	Conditional	0.831 (6)	30.7	< 0.001
Post	11.0 (13.1)	−1.1‐23.2				Marginal	0.831 (5)	23.8	< 0.001

* Models adjusted for job level, time working at hospital, and weekly workload.

By contrast, analysis of the Intervention Group (IG) revealed a statistically significant effect of the educational intervention on the general knowledge in EBP score. This positive effect was also confirmed when the ACE and Fresno test results were evaluated individually (Table 3).

**TABLE 3:** Linear Mixed Model for general knowledge in Evidence‐Based Practice (EBP) scores, ACE test, and Fresno Test, pre‐ and post‐intervention in Intervention Group (*n* = 18).

Variables	Mean (SD)	95% CI	% difference	F (df)	*P*	Model Fit	*R* ^2^ (df)	LRT *X* ^2^	*p*
General score (0%–100%)*									
Pre	36.3 (9.6)	31.5–41.1	19.1	54.2 (1)	< 0.001	Conditional	0.727 (11)	36.1	< 0.001
Post	55.4 (9.5)	50.7–60.1				Marginal	0.539 (10)	36.1	< 0.001
ACE test (0%–100%)*									
Pre	65.9 (13.9)	59.0–72.8	10.8	6.9 (1)	0.014	Conditional	0.277 (11)	15.5	0.16
Post	76.7 (9.2)	72.1–81.3				Marginal	0.277 (10)	15.5	0.12
Fresno test (0%–100%)*									
Pre	18.5 (11.8)	12.6–24.3	24.2	35.7 (1)	< 0.001	Conditional	0.631 (11)	29.8	0.002
Post	42.7 (14.9)	35.3–50.0				Marginal	0.481 (10)	29.8	< 0.001

* Models adjusted for educational level, job level, time working at the hospital, and weekly workload.

The model assessing general knowledge in EBP scores showed excellent explanatory capacity: the fixed effects of the intervention, adjusted for covariates, accounted for 53.9% of the total variance (marginal *R* ^2^ = 0.539), while the random effects (participants) contributed an additional 18.8% (conditional *R* ^2^ = 0.727). These findings indicate a strong model fit and high explanatory capacity, with 72.7% of the variance in EBP knowledge explained. The model's statistical robustness was further supported by the Likelihood Ratio Test (LRT), with LRT *χ* ^2^ = 36.1; *p* < 0.001, confirming that the model including the intervention provided a significantly better fit than the model without the intervention, reinforcing the effectiveness of the educational intervention (Table 3).

The model for the ACE test score, however, exhibited lower explanatory power (conditional *R* ^2^ = 0.277). Given that the marginal and conditional *R* ^2^ values were identical, it can be concluded that the random effects contributed no additional explanatory value–indicating that all explained variance stemmed from the fixed effects (intervention and covariates). This conclusion is supported by the non‐significant LRT result (*χ* ^2^ = 15.5; *p* > 0.05), suggesting that inclusion of random effects promoted no significant improvement in model fit. In this case, a repeated measures model including only fixed effects would likely have sufficed to detect the intervention's impact (Table 3).

The Fresno Test model exhibited excellent fit (conditional *R* ^2^ = 0.631), with the intervention accounting for 48.1% of the total variance (marginal *R* ^2^ = 0.481) and the random effects contributing an additional 15%, totaling 63.1% of the explained variance. These results reinforce the positive impact of the intervention on the knowledge assessed by this instrument. Moreover, the model incorporating the intervention showed a significantly better fit compared to the model without the intervention, as evidenced by the LRT (*χ* ^2^ = 29.8; *p* = 0.002), further supporting the suitability of the LMM for this analysis (Table 3).

**FIGURE 2:**
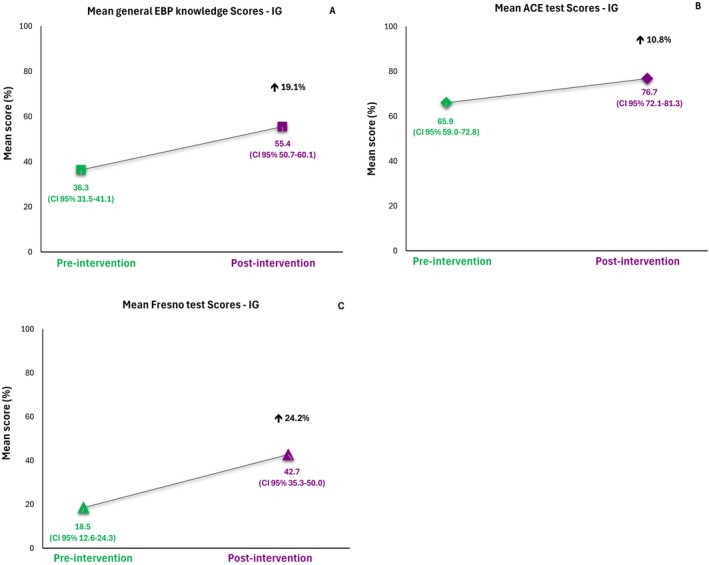
Graphical representation of increase in mean scores for general knowledge in EBP (A), ACE test (B), and Fresno test (C), pre‐ and post‐intervention in Intervention Group (*n* = 18).

The graphical results of the Linear Mixed Model (LMM) are presented in Figure. The model assessing the general knowledge in EBP score showed a 19.1% increase following the intervention compared to baseline (Figure 2A). Similarly, the ACE test model showed a 10.8% improvement (Figure 2B), while the Fresno test model showed a 24.2% increase (Figure 2C).

Results of the LMM analysis examining the interaction between Group (CG vs. IG) and Time (pre‐ vs. post‐intervention) revealed a statistically significant interaction effect for general EBP knowledge (*F* [1, 14.23] = value; *p* < 0.001) and for the Fresno Test (*F* [1, 13.37] = value; *p* = 0.001). No significant interaction effect was found for the ACE test (*F* [1, 1.59] = value; *p* = 0.22). For both general EBP knowledge and Fresno test scores, IG participants had higher scores than CG participants on both pre‐ and post‐intervention assessments. Additionally, pre‐intervention scores in the IG were significantly higher than those in the CG (*p* < 0.001, Bonferroni post hoc test).

### 3.3 Course Evaluation

**FIGURE 3:**
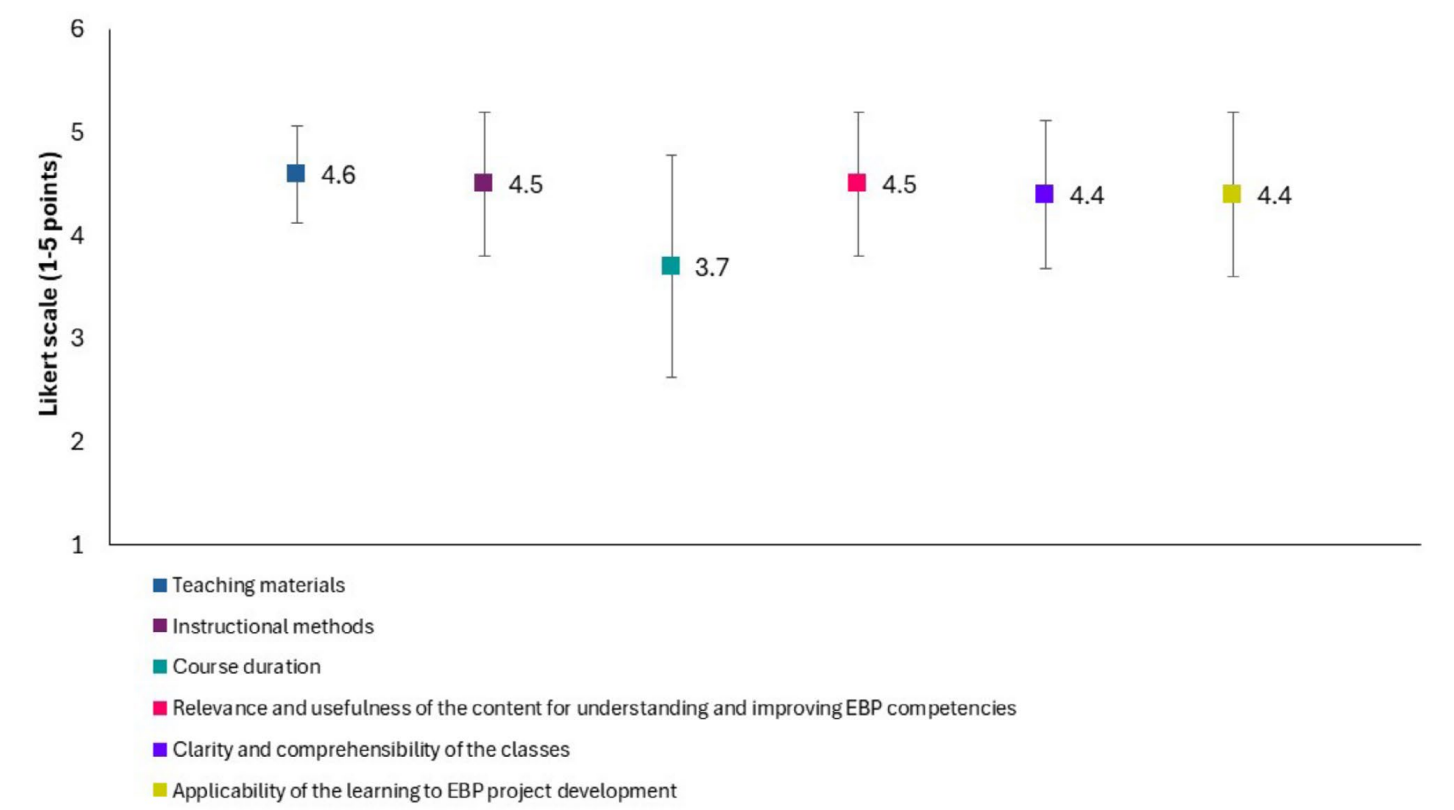
Participant perceptions of course and its applicability in development of EBP projects (*n* = 18).

Regarding participant perceptions of the course and its applicability to EBP project development, the surveys revealed high satisfaction. Teaching materials (mean = 4.6, SD = 0.47), instructional methods (mean = 4.5, SD = 0.70), relevance and usefulness of the content for understanding and improving EBP competencies (mean = 4.5, SD = 0.70), clarity and comprehensibility of the classes (mean = 4.4, SD = 0.72), and applicability of the learning to EBP project development (mean = 4.4, SD = 0.80) received the highest ratings. Conversely, the course duration received the lowest average rating (mean = 3.7, SD = 1.08), indicating greater variability in participant responses for this aspect (Figure 3).

## 4 Discussion

The findings of this randomized clinical trial demonstrate that an educational intervention focused on evidence‐based practice (EBP) significantly improved healthcare knowledge held by the professionals studied. Participants also rated the quality and applicability of the intervention highly, particularly regarding its usefulness for developing EBP projects. Furthermore, the group that received the intervention showed superior results compared to the control group at both pre‐ and post‐intervention assessments, including for overall EBP knowledge and performance on the Fresno Test. These results support the effectiveness of ongoing professional training in this area and highlight the importance of well‐structured, tailored educational strategies to enhance EBP competencies among healthcare professionals.

The application of a Linear Mixed Model allowed for a robust analysis by accounting for both fixed effects (such as the intervention and covariates) and participant heterogeneity. This approach yielded models with high explanatory power, a conditional *R* ^2^ of 72.7% for the overall score and 63.1% for the Fresno Test. The comprehensive methodological approach applied distinguishes this study from previous research, which typically employs simpler models that limit understanding of individual variability and the validity of conclusions. The current findings highlight the importance of considering participant heterogeneity when evaluating educational interventions, leading to a clearer understanding of their impact on EBP development.

Comparing the present results with the existing literature, the study by Mickan et al. (2019) also reported improvements in healthcare professionals' confidence and behavioral changes following training, although no significant gains in knowledge were observed, as measured by the Fresno Test–possibly due to the short duration of the intervention (eight hours). Conversely, Jeong et al. (2024) suggested that interventions lasting 20 to 24 h are more effective for developing EBP competencies, indicating that both duration and intensity are critical factors for successful outcomes.

In the present study, the robust statistical model revealed positive effects across the various assessment tools, including a 24.2% increase in EBP competencies on the Fresno Test. This improvement reinforces the effectiveness of the intervention, even when measured by more complex evaluation instruments. These findings align with literature emphasizing that longer, more intensive training programs are associated with greater skill acquisition in EBP (Tucker et al. 2022; Jeong et al. 2024).

For example, Melender et al. (2020) conducted a quasi‐experimental intervention lasting 21 h involving 48 healthcare professionals and social workers, using questionnaires developed by the authors. The results showed improvements in evidence‐based practice (EBP) knowledge (Friedman *p* < 0.01), participant confidence conducting database searches (Friedman *p* < 0.01), confidence reading scientific articles (Friedman *p* < 0.01), and in the use of resources such as Medic (Cochran *p* < 0.01), PubMed (Cochran p < 0.01), and CINAHL (Cochran *p* < 0.01). Furthermore, participants showed increased initiative to investigate work‐related topics (Cochran *p* < 0.01). However, no improvements were observed in the perceived importance of scientific knowledge (Friedman *p* > 0.05), in the use of scientific articles in clinical practice (Cochran *p* > 0.05), in conducting database searches during clinical practice with students (Cochran *p* > 0.05), or in the provision of thesis topics to students (Cochran *p* > 0.05). These findings may be attributed to the use of non‐validated instruments, which limits the sensitivity to detect more subtle changes (internal validity). These results underscore the importance of using validated instruments for the evaluation of educational interventions, as proposed in the present study.

Another relevant study was that conducted by Tucker et al. (2022), who implemented a five‐day (40 h) practical immersion intervention involving leaders of a cancer center, employing validated scales to assess knowledge, beliefs, competencies, and confidence in EBP. The course was delivered by an international institute for the teaching and dissemination of best practices in EBP, affiliated with the university where the cancer center is located. Mixed linear model analysis, adjusted for age, sex, educational level, job role, length of employment, and leadership experience, revealed significant improvements across all dimensions evaluated. These findings highlight the effectiveness of longer, more intensive interventions in promoting EBP competencies. The results also reinforce the importance of conducting studies that ensure high internal validity through the use of validated questionnaires, as well as high external validity by means of adequate sample size. Additionally, the need to control participant heterogeneity through appropriate statistical analyses is evidenced, where such approaches allow the positive impact of the proposed educational intervention to be shown effectively.

By contrast, shorter interventions, such as that of Tomotaki et al. (2024), which involved lectures and small‐group discussions, showed statistically significant improvements only in the research knowledge and skills subscale (repeated measures ANOVA *p* < 0.05). No statistically significant results were found in the practice, attitude, or practical knowledge and skills subscales, nor in total score on the Evidence‐Based Practice Questionnaire. These findings suggest that shorter interventions may be effective for addressing specific aspects of evidence‐based practice (EBP) but may not be sufficient to promote comprehensive changes in professional practice.

The authors also reported that the vast majority of participants were satisfied with the educational intervention, but many identified barriers to implementing EBP in clinical settings, such as job changes, excessive workload, and institutional priorities (Tomotaki et al. 2024). These barriers can limit the impact of educational strategies, underscoring the need for multifaceted, long‐term approaches. The literature further highlights that lack of knowledge, mentorship, and supportive infrastructure constitute major obstacles to effective implementation of EBP, as noted by Tucker et al. (2022), Sevy Majers and Warshawsky (2020), and Lunden et al. (2020). These authors emphasized that fostering an organizational culture conducive to evidence use requires that leaders hold not only technical knowledge but also leadership skills and confidence to promote continuous learning environments. Therefore, training programs that include mentorship, institutional support, and time dedicated to developing EBP projects are essential for consolidating sustainable change.

In line with these findings, the recent systematic review and meta‐analysis conducted by Hill et al. (2024), which included only randomized controlled trials, reinforced that, while evidence‐based healthcare educational interventions may lead to positive improvements in knowledge, skills, attitudes, and behaviors immediately following the intervention, these effects tend to diminish over time, with no evidence of long‐term effects beyond six months. The main educational interventions reviewed were delivered in person, although some studies employed online or blended approaches, with minimal differences in outcomes between delivery methods. The authors emphasized that all studies reviewed were rated as having a high risk of bias, with effect estimates graded as low to very low certainty, requiring caution in the interpretation of results. Additionally, the heterogeneity among studies in terms of pedagogical approaches, duration, and frequency of interventions limited the generalizability of the findings. The authors also highlighted the need for more methodologically rigorous studies, with larger sample sizes and long‐term follow‐up, to establish more effective and sustainable strategies (Hill et al. 2024).

Within a framework of online training, the quasi‐experimental study of Ramos‐Morcillo et al. (2024) showed that online educational programs (duration 72 h) can be effective in enhancing EBP competencies, particularly when adjusted for contextual and professional variables. These programs represent a viable alternative for expanding the reach of educational efforts, especially in settings where in‐person training is limited by logistical or resource constraints. However, it is essential that such interventions be accompanied by ongoing support, such as mentorship and practical application opportunities, in order to consolidate the changes promoted and overcome the barriers frequently encountered in clinical practice. Furthermore, institutional culture and leadership support likely play a role in participant engagement and course viability. The presence of supportive leadership, alignment with national EBP priorities, and a growing institutional focus on quality and safety can facilitate EBP implementation. These factors should be considered when replicating the intervention in other settings.

### 4.1 Implications for Practice

Based on the results obtained in this study, it can be inferred that personalized professional educational interventions targeting healthcare professionals–particularly in hospital settings–should be broadly disseminated and encouraged. This recommendation is timely, given that knowledge of evidence‐based practice (EBP) remains limited among many health professionals. Programs of this nature have the potential to enhance participant confidence in research skills and EBP knowledge, as well as the capacity for critical analysis of scientific literature. Consequently, these professionals can better contribute to improving the quality and safety of patient care.

However, such programs should not be offered in isolation, where continuity of educational initiatives is crucial to maintaining motivation, knowledge, and competencies. In addition, institutions should endeavor to provide follow‐up support for EBP projects, such as mentorship offered by qualified professionals to EBP groups, considering the complexity involved in developing and implementing these projects.

Furthermore, institutions should ensure adequate support by allocating protected time for professionals to engage in EBP project development, as the implementation of such practices demands a substantial time investment. In this regard, it can be concluded that educational interventions in EBP, when well‐designed, of sufficient duration, rigorously evaluated using validated instruments, and supported institutionally, can lead to significant improvements in healthcare professionals' knowledge, skills, and attitudes.

Thus, the integration of evidence‐based practice into the daily routine of healthcare services requires comprehensive strategies that involve continuous investment in training, infrastructure, and institutional policies. These strategies must promote lifelong learning, interdisciplinary collaboration, and the recognition of evidence use as a central component in clinical decision‐making.

Future programs could be enhanced by incorporating measures to assess the impact of EBP education on patient care and satisfaction. Gathering data on how professionals integrate patient preferences into their practice or evaluating patient outcomes related to EBP‐driven interventions could provide valuable insights into the real‐world effectiveness of these programs.

### 4.2 Strengths and Study Limitations

A key strength of this study lies in the implementation of an individualized educational program, tailored to the specific needs and profiles of the participants. This personalized approach contributed to a potentially more effective intervention. Furthermore, the impact of the educational intervention on EBP competencies among the healthcare professionals was evaluated through a randomized controlled trial, considered the gold standard for assessing the effectiveness of interventions.

In addition, the statistical robustness of the models used–demonstrated by likelihood ratio tests–highlighted the importance of correctly specifying the structure of random effects, with the full models outperforming their reduced counterparts. The indicators derived from the Linear Mixed Models confirmed good model fit and statistical consistency, reinforcing the validity of the findings. The favorable results from the likelihood ratio tests (LRT), for both overall score and the Fresno test, confirmed the appropriate inclusion of random effects, thus strengthening the reliability of the inferences drawn. These elements supported the reinforcement of evidence‐based educational strategies and demonstrated the potential of the methodological approach employed for evaluating complex interventions in healthcare settings.

The limited number of participants completing both assessments, particularly in the CG, reduced the statistical power of between‐group comparisons and limits the generalizability of our findings. To assess potential bias introduced by attrition, we compared demographic and professional characteristics of CG participants who completed both assessments with those who dropped out. No significant differences were found, indicating that losses were likely random and did not compromise the validity of the findings. Future studies may improve participant retention through strategies such as automated reminders, incentives for post‐test completion, or integrating assessments into the institutional training framework.

The main limitation of this intervention was the lack of longitudinal follow‐up after the course ended. The study included only one post‐intervention assessment, conducted immediately after the course, with no subsequent evaluations to determine whether the outcomes persisted over the medium or long term. Another limitation is that the study focused on knowledge and test performance without assessing how participants applied EBP in real‐world clinical practice. Future research should include follow‐up assessments of behavioral change and practical implementation of EBP.

Given that the intervention was conducted in a single private hospital, caution is warranted when generalizing findings to public or academic settings. Differences in institutional culture, resource availability, and organizational support may affect implementation and outcomes.

To facilitate adaptation in other contexts, institutions should carefully consider their unique needs and resources. Strategies for adapting the intervention might include tailoring the content to local guidelines, adapting the duration and intensity of the course to fit available resources, and incorporating online components to increase accessibility. Furthermore, assessing the existing level of EBP competency among staff and aligning the intervention with organizational priorities can improve its effectiveness.

Beyond education, institutions can foster EBP adoption by creating a supportive culture through leadership, mentorship, and protected time. Leaders should champion EBP by communicating its value and providing resources for its implementation. Mentorship programs can pair experienced EBP practitioners with novices to provide guidance and support. Moreover, allocating protected time for professionals to engage in EBP project development is essential for overcoming the barriers often encountered in clinical practice. By addressing these factors, institutions can create a sustainable environment that supports the long‐term integration of EBP into clinical care.

## 5 Conclusion

This personalized professional educational intervention had a positive and significant effect on Evidence‐Based Practice (EBP) knowledge among healthcare professionals in the intervention group in a private tertiary general hospital. The results of the randomized controlled trial showed superior effects in the intervention group compared to the control group after controlling for potential biases and baseline differences between groups and ensuring the outcomes observed were indeed attributable to the intervention.

Moreover, the statistical models indicated the intervention adequately captured the impact of the strategy and variability among participants. This strengthens the evidence supporting that the approach can effectively enhance EBP competencies among healthcare professionals.

## Conflicts of Interest

The authors declare no conflicts of interest.

## Supporting information


**Supplementary Table 1:** Work units of healthcare professionals (*n* = 25).

## Data Availability

The data that support the findings of this study are available on request from the corresponding author. The data are not publicly available due to privacy or ethical restrictions.
