# Supplementary material for: Transforming Hospital Care: Impact of an Evidence‐Based Practice Course on Healthcare Professionals' Competencies in a Randomized Clinical Trial
Source: Worldviews Evid Based Nurs. 2026 Apr 1;23(2):e70130. doi: 10.1111/wvn.70130 (PMC13044389; doi:10.1111/wvn.70130)
Supplement: Supplementary file 1 — Supplementary Table 1: Work units of healthcare professionals (n = 25). [file WVN-23-0-s001.docx]

**Supplementary Table 1 –** Work units of healthcare professionals (n = 25).

| **Work Unit** | **CG** | **IG** |
| --- | --- | --- |
|  | **n (%)** | **n (%)** |
| Oncology outpatient clinic | 1 (14.3%) | 1 (5.6) |
| Oncology outpatient clinic, Diagnostic and Therapeutic Support Service, Specialty Medical Center | 0 (0) | 1 (5.6) |
| Surgical Center | 0 (0) | 1 (5.6) |
| Corporate sector | 0 (0) | 1 (5.6) |
| Pharmaceutical care | 0 (0) | 1 (5.6) |
| Emergency Department | 1 (14.3%) | 1 (5.6) |
| Clinical Practice Department | 0 (0) | 1 (5.6) |
| High Reliability/Quality Department | 2 (28.6) | 2 (11.1) |
| Diagnostic and Therapeutic Support Service | 0 (0) | 1 (5.6) |
| Hospital Infection Control Service | 1 (14.3%) | 1 (5.6) |
| Inpatient unit | 0 (0) | 1 (5.6) |
| Inpatient unit, Adult and Pediatric Intensive Care unit, Emergency Department, Surgical Center, Step-down unit, Oncology, Oncology Outpatient clinic, Specialty Medical center | 0 (0) | 1 (5.6) |
| Adult Intensive Care unit | 2 (28.6) | 3 (16.7) |
| Adult and Pediatric Intensive Care unit | 0 (0) | 1 (5.6) |
| Step-down unit | 0 (0) | 1 (5.6) |
